# Coriolis: enabling metagenomic classification on lightweight mobile devices

**DOI:** 10.1093/bioinformatics/btad243

**Published:** 2023-06-30

**Authors:** Andrew J Mikalsen, Jaroslaw Zola

**Affiliations:** Department of Computer Science and Engineering, University at Buffalo, Buffalo, NY 14260, United States; Department of Computer Science and Engineering, University at Buffalo, Buffalo, NY 14260, United States

## Abstract

**Motivation:**

The introduction of portable DNA sequencers such as the Oxford Nanopore Technologies MinION has enabled real-time and in the field DNA sequencing. However, in the field sequencing is actionable only when coupled with in the field DNA classification. This poses new challenges for metagenomic software since mobile deployments are typically in remote locations with limited network connectivity and without access to capable computing devices.

**Results:**

We propose new strategies to enable in the field metagenomic classification on mobile devices. We first introduce a programming model for expressing metagenomic classifiers that decomposes the classification process into well-defined and manageable abstractions. The model simplifies resource management in mobile setups and enables rapid prototyping of classification algorithms. Next, we introduce the compact string B-tree, a practical data structure for indexing text in external storage, and we demonstrate its viability as a strategy to deploy massive DNA databases on memory-constrained devices. Finally, we combine both solutions into Coriolis, a metagenomic classifier designed specifically to operate on lightweight mobile devices. Through experiments with actual MinION metagenomic reads and a portable supercomputer-on-a-chip, we show that compared with the state-of-the-art solutions Coriolis offers higher throughput and lower resource consumption without sacrificing quality of classification.

**Availability and implementation:**

Source code and test data are available from http://score-group.org/?id=smarten.

## 1 Introduction

The current portable DNA sequencers, such as the Oxford Nanopore Technologies (ONT) MinION (Oxford Nanopore Technologies 2022), allow for DNA sequencing directly in the field using relatively fast and easy wet lab protocols. Indeed, in recent years portable sequencers have aided with *in situ* diagnosis of diseases including COVID-19 (Oxford Nanopore Technologies 2022), Ebola (Quick et al. 2016), and Zika (Faria et al. 2016), and have been successfully deployed in remote locations such as the Antarctic (Johnson et al. 2017), the rainforests of Ecuador (Pomerantz et al. 2018), and even the International Space Station (Castro-Wallace et al. 2017). For some time now, researchers and practitioners have been envisioning the Internet of Living Things (Waltz 2017): the idea that anyone will be able to sequence and analyze the DNA of anything anywhere. If achieved, this would open new possibilities in, e.g. rapid medical diagnosis, tracking infectious diseases, biological threat detection, and microbial community monitoring in remote locations (Faria et al. 2016; Quick et al. 2016; Quince et al. 2017; Walter et al. 2017; Gardy and Loman 2018; Ko et al. 2018). Nevertheless, to realize this vision, portable DNA sequencers must be complemented with real time and in the field DNA analysis. As many case studies have remarked and demonstrated, to be truly portable, the analysis must be performed locally and on low-energy and small form-factor mobile devices (Quick et al. 2016; Ko et al. 2018; Oliva et al. 2020). Realistic in the field deployments have little, if any, network connectivity, and given the massive volumes of data streamed by MinION sequencers (currently up to 50 GB per flow cell; Oxford Nanopore Technologies 2022), it is impossible to delegate analysis to remote data centers. Moreover, using workstations or high-end laptops is equally impractical, as they are expensive, difficult to transport, and still require a substantial and steady supply of energy (see Ko et al. 2018 for a more detailed survey on mobile DNA sequencing).

In the standard mobile DNA sequencing setup, a portable sequencer is attached to a mobile host device, e.g. a mobile phone, a supercomputer-on-a-chip (SCoC), etc. The workflow then involves fast DNA library preparation protocols and real-time sequencing. The obtained raw signals from the sequencer are basecalled and the resulting reads are passed to some analytics software running on the host device. While technically any type of analysis could be used, usually the most desired is metagenomic classification. In this setup, to make the data produced by the sequencer immediately actionable, the host device must perform the analysis in step with sequencing, i.e. in the field and in real-time.

However, the software tools for performing metagenomic classification are both compute and memory intensive, owing to the massive reference databases on which they operate, and have been designed for setups where resources such as compute, memory, energy, network, and storage are not a concern. But ample resources are hardly ever available in mobile environments, even if we consider highly capable SCoC devices, e.g. the ONT MinIT or MinION Mk1C (Oxford Nanopore Technologies 2022). This is further complicated by the fact that the analytics software (i.e. the metagenomic classifier) must share resources with the computationally expensive basecaller as well as the sequencer control software. Consequently, the current software tools are incapable of performing DNA analysis on mobile devices (Oliva et al. 2020).

In this article, we propose methods for metagenomic classification designed bottom-up to tolerate the conditions of mobile environments. Our contributions are 2-fold. First, we provide abstractions for metagenomic classification that enable both more effective management of scarce resources on mobile devices and the rapid prototyping of metagenomic classification algorithms. We implement these abstractions in the form of our programming model and software framework, System for Mobile Analysis in Real-Time of Environment (SMARTEn), and demonstrate their merit by implementing Coriolis—a mobile metagenomic classification tool. Second, we introduce the compact string B-tree (CSBT), a practical out-of-core text index, as a strategy to effectively manage genomic reference databases on lightweight mobile devices. By virtue of I/O optimality, the CSBT can serve massive databases on low-memory systems while retaining extremely fast and energy efficient searches. Through experiments with actual mobile devices and real-world MinION sequencing data, we show that by coupling our programming model with CSBTs we can deliver a metagenomic classifier that outperforms and out scales the state-of-the-art tools when operating on lightweight devices.

## 2 SMARTEn programming model

To design a metagenomic classifier suited for mobile environments, we must completely rethink the current classification software architecture (Ko et al. 2018). Efficiently processing massive volumes of data in mobile settings requires careful management of sparse resources such as compute, memory, energy, network, and storage. This in turn calls for a modular design to isolate the key functionalities and thus simplify their management, optimization, and interfacing with each other and with hardware or other applications. While a number of works have attempted to deploy bioinformatics tools in mobile setups (e.g. Merelli et al. 2018; Milicchio et al. 2018; D’Agostino et al. 2019; Grzesik and Mrozek 2021), the emphasis has been primarily on testing the limits of the existing software in such deployments. The current bioinformatics tools remain monolithic and ill-suited for mobile environments (Oliva et al. 2020). To address this challenge, we propose an abstract representation of the metagenomic classification workflow which we realize in a dedicated programming model. The use of suitable and basic abstractions has been previously demonstrated to significantly improve the efficiency, maintainability, and development speed of software (Dean and Ghemawat 2004; Massie et al. 2013). We wager that our proposed abstractions, however simple, are powerful enough to decompose and optimize virtually any metagenomic classification algorithm.

### 2.1 Programming model and API

**Figure 1.**
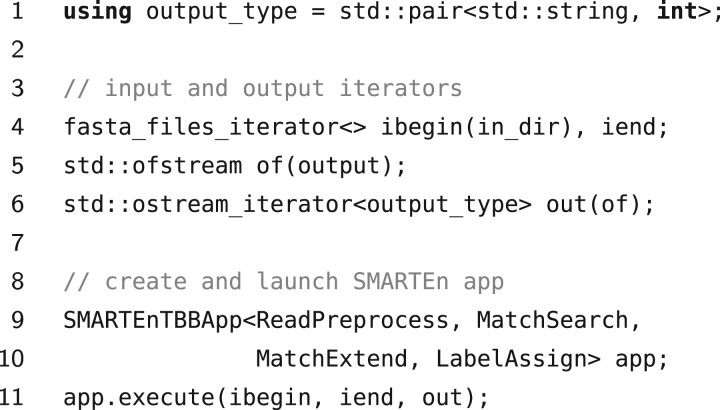
Sample code instantiating a SMARTEn application. The four types passed to SMARTEnTBBApp (i.e. the SMARTEn runtime) are defined by the end-user, following a well-defined and documented API, to express the classification algorithm.

The SMARTEn programming model provides C++ constructs that enable programmers to focus on the key algorithmic components when implementing a metagenomic classifier without worrying about extraneous low-level details, e.g. how input, intermediate, and output data is managed and how the actual execution is carried by the underlying hardware. From the programmer’s perspective, an algorithm expressed in SMARTEn transforms a collection of key-value pairs representing a sequence of input reads into a mapping of each input read to a set of taxonomic labels (i.e. a set of taxonomic assignments). Here, a key-value pair representing a read denotes the read’s identifier along with its content (e.g. a string of ACGTs or a MinION signal). Users express this transformation by defining four procedures (represented as types) corresponding to the stages of the abstract workflow: ReadPreprocess, MatchSearch, MatchExtend, and LabelAssign. To this end, the users are provided with a minimal set of well-documented functions that they can use to interact with the SMARTEn runtime (SMARTEn 2023). The user defined types, which follow a predefined API, are then combined with the SMARTEn provided types representing the actual runtime system to obtain the final application, which takes over the execution of the classifier on a sequence of input reads (see Fig. 1). The user defined types, without any changes, can be combined with any SMARTEn runtime (currently we provide two runtimes, one offering basic sequential execution, and one providing automatic end-to-end parallelization) to automatically benefit from the runtime’s features.

**Figure 2.**
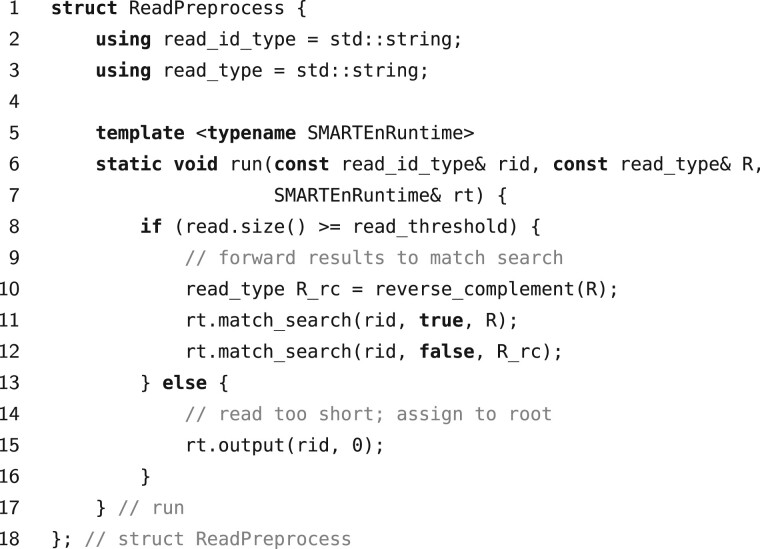
Sample implementation of a ReadPreprocess type. The method run is user specified, while the SMARTEn type SMARTEnRuntime abstracts the runtime and the function match_search lets the user pass data to the MatchSearch stage.

The first stage, ReadPreprocess, performs the initial preprocessing of a given input read, e.g. filtering out too short or too erroneous reads, computing the reverse complement of a read, or decomposing a read into its *k*-mer spectrum. The SMARTEn runtime automatically executes this stage once per input read. The stage takes as its input a single $\left(r_{id},R\right)$ pair, where *r_id_* is the read’s unique identifier and *R* its content. By calling the match_search, match_extend, label_assign, and output functions (provided by the runtime), this stage can forward its results to any of the subsequent stages (typically to MatchSearch) and can output taxonomic assignments. As an example, Fig. 2 shows a code snippet of the ReadPreprocess type we implemented as part of our classifier in Section 4.

The second stage, MatchSearch, performs the initial searching for occurrences of the read in the user-provided reference database to identify potential matches. Typically, this involves enumerating significantly matching positions or looking for occurrences of fixed-sized words (e.g. *k*-mers) shared between the read and the sequences in the reference database. This stage takes as its input triplet $\left(r_{id},q_{id},Q\right)$, where $\left(q_{id},Q\right)$ represents a match search query, usually a processed read from ReadPreprocess, and *r_id_* denotes the identifier of the read used to generate the initial query during ReadPreprocess. This stage can forward results to itself, any stage that follows it, and can output taxonomic assignments. To give complete freedom to the end user, we make no assumptions about the representation of the reference database, and can expect that the resulting computational task will still be handled efficiently.

The third stage, MatchExtend, is an optional stage intended for scenarios where the algorithm may need to further inspect matches identified during MatchSearch (a classic example is expanding an initial exact match into a local alignment). Like MatchSearch, MatchExtend takes a triplet $\left(r_{id},q_{id},Q\right)$, but the types of *q_id_* and *Q* do not need to be the same as those in MatchSearch. The MatchExtend stage can forward results to itself, LabelAssign, and output taxonomic assignments.

The fourth and final stage, LabelAssign, does the final assignment of taxonomic labels to an input read. Unlike the previous stages, LabelAssign takes a pair $\left(r_{id},L\right)$, where *r_id_* is the identifier of the read being classified (assigned), and *L* is an unordered sequence of all values forwarded to LabelAssign by previous stages for the given *r_id_*. The programmer can use any type for the elements of *L*, but it will often be a tuple consisting of the assigned taxonomic identifier along with the evidence supporting the assignment (e.g. positions and lengths of matching read fragments). This stage can only output taxonomic assignments. It will typically involve inspecting all assigned labels and determining which should be used for the final classification of the read, usually by some form of traversal and scoring over the taxonomic tree.

With this decomposition, the programmer only expresses the high-level classifier stages. All low-level details, such as data flow, execution of the resulting computational tasks, etc., are transparently managed by the SMARTEn runtime system. Our current implementation of the runtime focuses on automatic parallel execution for shared memory systems. A main thread iteratively extracts and batches reads from an input stream, and once the size of the batch reaches a certain threshold and sufficient compute resources are available, creates a computational task that is dispatched by the Intel TBB platform. The tasks are handled by the TBB scheduler, which assigns them to executing threads. The runtime is also complemented with multiple coding facilities, including iterators and file system monitors to interact with different inputs, e.g. FASTA, FASTQ, and FAST5 files, either stored in external storage or streamed in real-time (e.g. by basecalling software). Collectively, the runtime and tools can be used to rapidly develop and deploy fully functional and efficient metagenomic classifiers.

While our proposed decomposition of the classification process may seem simple, it is surprisingly extensive and captures the current popular tools operating both in the string space and, more recently, in the MinION signal space (Zhang et al. 2021). A quick review shows that tools including MegaBLAST (Zhang et al. 2000), Pathoscope (Hong et al. 2014), Kraken (Wood et al. 2019), Centrifuge (Kim et al. 2016), SigMap (Zhang et al. 2021), and many others can be logically decomposed into this four-stage workflow. This decomposition has the critical advantage that it separates stages having significantly different properties (e.g. fast preprocessing versus I/O and memory intensive searching) into individual entities whose execution can be optimized and specialized individually. Furthermore, since the executing runtime is completely separated from the actual classification algorithm, the same classifier can be optimally deployed on different hardware platforms just by changing the runtime.

## 3 The compact string B-Tree

One signature characteristic of metagenomic classification is the use of massive reference databases. Searching the reference database is the most expensive part of the classification process, even when utilizing dedicated high-speed indexes. Reference databases are growing exponentially in size and can already be as large as several TB (Stephens et al. 2015). Furthermore, their rate of growth far outpaces improvements to RAM memory, which, because it is expensive and power hungry, is available in only limited quantities to mobile devices. Thus, for practical applications, the current and future mobile devices, even highly capable SCoCs, have insufficient amounts of main memory (at present only a few GB) in which to store the indexes. Even though some tools try to address this limitation via memory-mapping techniques, e.g. Centrifuge, this solution results in slow classification and high energy usage caused by excessive I/O operations.

The strategy that is frequently adopted when dealing with limited memory setups is to utilize data structures explicitly designed for external storage. In this work, we build upon Ferragina and Grossi’s string B-tree (SBT) (Ferragina and Grossi 1999), an external memory (EM) data structure for exact pattern matching. SBTs are based on B-trees of suffixes where each B-tree node is embedded with an auxiliary Patricia trie over its keys. However, this entails that SBT nodes must be embedded with significant amounts of auxiliary data. Consequently, SBTs have high storage requirements and are relatively slow in practice. To address these limitations, we propose the CSBT, a practical variant of the SBT. The CSBT is I/O optimal under the EM model (Vitter 2008), which measures efficiency in terms of the number of I/O operations, and is highly suited for indexing text in low-memory environments, as it is space efficient, alphabet independent, and easily generalizes to indexing multiple strings.

The CSBT is a B-tree of suffix offsets, where all unique suffixes are stored in the leaves and replicated at the higher levels of the tree. For a block size of *B*, every node’s branching factor is b=Θ(B). Every leaf stores *b* suffixes, while every parent stores each child’s rightmost suffix, resulting in at most *b* keys per node. To efficiently handle multiple texts, the CSBT stores the positions of duplicate suffixes separately from the tree, somewhat resembling a generalized suffix array. Each child pointer of a leaf points to an array containing all positions at which the corresponding suffix occurs, akin to how a B${}^{+}$-tree stores its keys and values. These arrays can be stored either contiguously or in linked Ω(B) chunks. We can also apply application specific optimizations by replacing these duplicate positions with other values, e.g. for metagenomics, the identifiers of the reference sequences containing the positions or even their LCA in the taxonomic tree. To obtain the optimal I/O bound, we embed each node with a compact index (described in Section 3.1) used to identify the successor of a pattern *P* among the keys of the node. This data structure must take *O*(*B*) space and must identify *P*’s successor using $O\left(\frac{|P|-\ell }{B}\right)$ I/Os given the knowledge that the node stores some suffix sharing an ℓ-length prefix with *P*. In addition to the embedded index, we also store in each node a value denoted *LCP*(1): the longest common prefix between the first string in the current node and the last string in the previous node on the same tree level. All data structures presented here can be easily constructed in *O*(*N*) time, where *N* is the text size. Because in practice index construction is a one-time effort that can be easily delegated to high-end servers, we concern ourselves primarily with the I/O complexity of querying the index.

Searching the CSBT involves first descending the tree to identify *P*’s lexicographic position among all suffixes, then scanning the leaves to report all occurrences of *P*. At each level of the search, we maintain ℓ, the length of the longest common prefix between *P* and the suffixes of the previous node (initially ℓ=0). If *P*’s successor in the previous node shared with it an ℓ-length prefix, or if LCP(1)≥ℓ, then the current node must contain a suffix that shares an ℓ-length prefix with *P*. Thus, when searching for *P*’s successor in the current node (using the embedded index), we avoid comparing the first ℓ characters of *P*. If this is not the case, then *P*’s lexicographic position among all suffixes must reside in the current node’s leftmost descending leaf, so we can complete the descent without comparing the remaining characters of *P*. This totals to $O\left(\frac{P}{B}+{\;\text{log}\;}_{B}N\right)$ I/Os to descend the tree. Next, to scan the leaves and report all *K* occurrences of *P*, we perform $O\left(\frac{K}{B}\right)$ I/Os. Thus, the final I/O complexity is $O\left(\frac{P+K}{B}+{\;\text{log}\;}_{B}N\right)$, which is asymptotically optimal under the comparison model (Ferragina and Grossi 1999).

### 3.1 Patricia arrays

The Patricia array is our compact encoding of a Patricia trie which achieves a space-efficient representation of CSBT nodes. As such, it can be seen as a generalization of the distinction bit array proposed by Ferguson (1992). We define a Patricia array as a 4-tuple of arrays S, *LCP*, *C_L_*, and *C_R_*, where S is the lexicographically sorted array of indexed strings (represented as pointers), *LCP* is the array of the longest common prefixes between successive strings in S, and *C_L_* and *C_R_* are the arrays of left and right mismatched characters, respectively. Formally, we have that $S=\langle S_{1},S_{2},\ldots ,S_{\left|S\right|}\rangle ,\;\mathrm{LCP}(i)=\mathrm{lcp}(S_{i-1},S_{i}),\;C_{L}(i)=S_{i-1}(\mathrm{LCP}(i)+1)$, and $C_{R}(i)=S_{i}(\mathrm{LCP}(i)+1)$, where *LCP*, *C_L_*, and *C_R_* are defined for 2≤i≤|S|. We conjecture that only *C_R_* is necessary to conduct the desired queries, but using both in the algorithms simplifies the proofs, has only a marginal space overhead (|S| additional characters), and is typically free in practice due to memory alignment and padding (e.g. padding the structure to align with the OS page size).

To ensure *C_L_* and *C_R_* are always defined, we assume that S is prefix free. When searching for a pattern *P*, we also assume that no *S_i_* is a prefix of *P*. Both these assumptions can be removed if we explicitly store the lengths of each *S_i_*, but they are easy to guarantee in practice by appending a special termination character to each *S_i_*. In the context of the CSBT, we will have |S|=Θ(B). Thus, since the Patricia array takes Θ(|S|) space, it will take *O*(*B*) space in a CSBT node. Unlike the Patricia trie, which requires storing an entire tree of at most 2·|S|−1 nodes, each containing suffix offsets, node pointers, edge labels, and suffix lengths, the Patricia array stores only four arrays, making it much more compact. Furthermore, its space complexity is independent of the alphabet size |Σ| and remains Θ(|S|) even if |Σ|=ω(1) (compared with Θ(|Σ|·|S|) for a Patricia trie).

Like Patricia tries, the Patricia array’s search algorithm consists of two phases. In the first phase, we identify some *S_j_* in S that has the maximum longest common prefix with *P*. In the second phase, we correct the position of *j* to be *P*’s successor in S. The first phase is given by Algorithm 3.1. The algorithm starts by assuming *j *=* *1, and adjusts *j* as it scans S sequentially from left to right. If we encounter an *S_i_* for which $P(\mathrm{LCP}(i)+1)=C_{R}(i)$, then we assign *i* to *j* and continue. Otherwise, we skip all entries in S until we find some *S_k_* such that LCP(k)≤LCP(i), after which we resume scanning.

Algorithm 1. Blind-Search(*P*)

1: j←1

2: ℓ←0

3: **for**i←2 up to |S|**do**

4:  **if**i−1=j or LCP(i)≤ℓ**then**

5:   ℓ←LCP(i)

6:   **if**$\ell <|P|\;and\;P(\ell +1)=C_{R}(i)$**then**

7:    j←i

8: **return** *j*

We can think of the algorithm as traversing a virtual Patricia trie, where the current *LCP*(*i*) corresponds to the current virtual node in the trie and each *S_i_* to a leaf node. The algorithm attempts to find a matching virtual path from the current node down to the leaves, retrying from higher levels in the virtual trie when it fails. The correctness of Algorithm 3.1 is stated formally in Theorem 1 (we provide proofs for all theorems in Supplement 1).

Theorem 1. Blind-Search(*P*) *returnsrsuch that*$\mathrm{lcp}(P,S_{r})=\max\{\mathrm{lcp}(P,S_{k})|1\leq k\leq |S|\}$.

Algorithm 2 starts by executing the first phase on line 1 and then begins the second phase on lines 2–20. In the second phase, we use the *S_j_* identified in the first phase to find *P*’s lexicographic position among the strings of S given that we know *P* and some string in S share an ℓ-length prefix. The algorithm returns two values: *j* denoting the position of *P* and $\ell \prime =\mathrm{lcp}(P,S_{j})$. First we compute $\mathrm{lcp}(P,S_{j})$ and determine if *P* comes before or after *S_j_*. We then scan either leftward or rightward of *j* to identify *P*’s lexicographic position. The correctness is stated in Theorem 2, and the I/O complexity in Theorem 3.

Theorem 2. *For alphabet Σ, given* $P\in \Sigma^{*}$and ℓ∈N∪{0}*such that no* $S_{i}\in S$*is a prefix of P and* $\ell \leq \max\{\mathrm{lcp}(P,S_{k})|1\leq k\leq |S|\}$*, Successor(*P,ℓ*) returns* (j,ℓ′)*such that* $j=\min\{1\leq k\leq |S|+1|k>|S|\vee P\leq S_{k}\}$*and* $\ell \prime =\max\{\mathrm{lcp}(P,S_{k})|1\leq k\leq |S|\}$.

Theorem 3. *Assume that* S*, LCP, C_L_, and C_R_ reside in internal memory and that each* $S_{i}\in S$*is represented as a pointer to some string stored in external memory. Given* $P\in \Sigma^{*}$such that no $S_{i}\in S$*is a prefix of P and* $\ell \leq \max\{\mathrm{lcp}(P,S_{k})|1\leq k\leq |S|\}$*, Successor(*P,ℓ*) takes* $O\left(\frac{|P|-\ell }{B}\right)$*I/Os.*

Because Patricia arrays answer the same queries as Patricia tries (Theorem 2) at the same I/O complexity (Theorem 3), they are an I/O optimal replacement for Patricia tries. Since Patricia arrays are also much smaller, they significantly reduce the storage requirements for the CSBT compared with the SBT. Moreover, they make the CSBT considerably faster in practice, since many more suffixes can be packed into a CSBT node than can be into an SBT node of the same size. With these improvements, we can build a metagenomic classifier based on exact pattern matching that remains very efficient even when there is very limited memory available, making the CSBT a perfect fit for indexing massive reference databases on mobile devices.

### 3.2 CSBT implementation

We implemented our CSBT data structure as a C++ library available from SMARTEn (2023). In our current implementation, the CSBT is stored on disk as a contiguous sequence of CSBT nodes whose access is abstracted by multiple, interchangeable memory managers. Our basic memory manager uses memory-mapping and thus delegates all low-level access operations to the operating system. Despite the fact that memory-mapping deprives us of fine-grained control over memory, the I/O-optimal nature of CSBTs allows it to operate efficiently even though the operating system may be unable to identify the hot working set of CBST nodes. To further reduce the CSBT’s size, we implemented a memory manager that performs on-the-fly compression and decompression of CSBT nodes. The manager uses the lightweight LZ4 algorithm (Collet 2022) and reduces node sizes by 26%, allowing us to load more data per I/O step, thus virtually expanding our cache.

## 4 Mobile metagenomic classifier

To enable metagenomic classification on mobile devices, we prototyped a complete metagenomic classifier using SMARTEn. Our classifier, called Coriolis, is based on exact pattern matching and closely resembles Centrifuge (Kim et al. 2016). Coriolis uses CSBTs to store its reference database indexes, which enables it to efficiently perform exact pattern matching in cases where the reference database is too large to fit into the limited main memory of a mobile device. The primary motivation for using exact pattern matching over the typically faster *k*-mer based matching (e.g. Kraken, Wood et al. 2019) is that exact pattern matching tends to be more error-resilient and provides higher sensitivity, especially for noisy and long MinION reads (Leidenfrost et al. 2020; Lu et al. 2022; Sczyrba et al. 2017). Below we provide the pseudocode expressing Coriolis via the individual SMARTEn stages.

Algorithm 2. Successor(P,ℓ)

1: j←blind-search(*P*)

2: $\ell \prime \leftarrow \ell +\mathrm{lcp}\left(P[\ell +1..|P|],S_{j}[\ell +1..|S_{j}|]\right)$

3: **if**|P|=ℓ′**then**

4:  **while**$j>1\;and\;\mathrm{LCP}(j)\geq \ell^{\prime }$**do**

5:   j←j−1

6: **else**

7:  c←P(ℓ′+1)

8:  **if**$c<S_{j}(\ell \prime +1)$**then**

9:   **while**$j>1\;and\;\mathrm{LCP}(j)\geq \ell^{\prime }$

    $and\;(\mathrm{LCP}(j)>\ell^{\prime }or\;c<C_{L}(j))$**do**

10:    j←j−1

11:  **else**

12:   j←j+1

13:   **while**j≤|S| and LCP(j)≥ℓ′

      $and\;(\mathrm{LCP}(j)>\ell^{\prime }or\;c>C_{R}(j))$**do**

14:    j←j+1

15: **return**(j,ℓ′)

Algorithm 3. Read- Preprocess(*r_id_*: str, *R*: str)

1: **if**$|R|\geq t_{\mathrm{read}}$**then**

2:  $\bar{R}\leftarrow$reverse-complement(*R*)

3:  match_search(*r_id_*, **true**, *R*)

4:  match_search(*r_id_*, **false**, $\bar{R}$)

5: **else**

6:  output($r_{id},0$) ◃ assign root of taxonomic tree as label

The pseudocode of Read-Preprocess is given in Algorithm 2. The procedure simply filters out too-short reads (line 1), and forwards data to Match-Search to initiate read classification (lines 3-4). Here we filter out all input reads shorter than the threshold *t_read_*, which by default is *t_read_* = 22, and we issue queries for both the actual read and its reverse complement. The match search query identifier (the second argument to match_search, denoted by *q_id_* in Section 2) distinguishes *R* from $\bar{R}$ in the subsequent stages. Finally, too short reads are immediately marked as unclassified and assigned the label of the taxonomic tree’s root (line 6).

Algorithm 4. Match-Search(*r_id_*: str, *q_id_*: bool, *Q*: str)

1: i←0

2: **while**$|Q|\geq i+t_{\mathrm{match}}$**do**

3:  (lcp,M)←Index.findQ[i..|Q|−1] ◃ find matches *M*

4:  **if**$\mathrm{lcp}\geq t_{\mathrm{match}}$**then**

5:   **for**m∈M**do** ◃ iterate over all matching positions *m*

6:    match_extend(*r_id_*, *q_id_*, (*m*, *lcp*))

7:  i←i+lcp+1

The match search queries generated by read preprocessing are handled by the Match-Search stage, described in Algorithm 3. Here, *q_id_* and *Q* specify the query read. Using a text index Index representing our reference database (a CSBT by default), the algorithm starts by matching characters in *Q* with the reference strings until encountering a mismatch (line 3). If the matching substring is of length at least *t_match_* (line 4), the algorithm creates a match_extend query for each matching position *m* in the reference database (lines 5-6). By default, *t_match_* = 16. Match extend queries take the form of tuples (*m*, *lcp*), where *m* is the position of the match in the reference database and *lcp* is the length of the match. The algorithm then continues matching *Q* from the position after the mismatched character (line 7), repeating this process until exhausting *Q* (line 2).

In the Match-Extend stage, given in Algorithm 5, we convert the position *m* in the reference database to the taxonomic identifier of the matching sequence. This match serves as a piece of supporting evidence to assign the taxonomic identifier *t_id_* to the query read identified by *r_id_*. With this information, we create a label_assign call (line 2) that takes the form of triplet $\left(q_{id},t_{id},\ell \right)$, where *q_id_* is a boolean denoting if the match was between the actual read or its reverse complement, *t_id_* is the taxonomic identifier of the reference sequence where the match occurred, and ℓ is the length of the match. We can interpret this call as a statement that the read *r_id_* may be classified with label *t_id_* because it has a match of length ℓ.

If we look at Algorithms 4 and 5 we can see that for a single input read, we may have multiple significant matches between a substring of the read and the reference database, resulting in multiple calls to label_assign. Thus, the job of the label assign stage, described by Algorithm 6, is to aggregate this supporting evidence, delivered by the calls to label_assign, into the final assignment of taxonomic labels (i.e. taxonomic assignment).

Algorithm 5. Match- Extend(*r_id_*: str, *q_id_*: bool, (*m*, ℓ: int))

1: $t_{id}\leftarrow$position-to-taxid(*m*)

2: label_assign(*r_id_*, $\left(q_{id},t_{id},\ell \right)$)

Algorithm 6. Label- Assign(*r_id_*: str, *L*: iterable)

1: H←∅ ◃ scores for forward matches

2: $\bar{H}\leftarrow \emptyset$ ◃ scores for reverse complement matches

3: **for**$(q_{id},t_{id},\ell )\in L$**do**

4:  **if**$q_{id}=t\mathrm{rue}$**then**

5:   $H[t_{id}]\leftarrow H[t_{id}]+{\left(\ell -\ell_{\min}\right)}^{2}$ ◃ Centrifuge’s scoring

6:  **else**

7:   $\bar{H}[t_{id}]\leftarrow \bar{H}[t_{id}]+{\left(\ell -\ell_{\min}\right)}^{2}$

8: $s_{\max}\leftarrow \max\{s^{\prime }|\exists_{t}(t,s^{\prime })\in H\cup \bar{H}\}$

9: $M\leftarrow \{t|(t,s_{\max})\in H\cup \bar{H}\}$ ◃ find all labels with the max score

10: P←∅ ◃ parent labels to replace assigned labels

11: **while**$|M|+|P|>t_{\mathrm{label}}$**do** ◃ reduce number of assigned labels

12:  $p\leftarrow \mathrm{choose}\;p\in \text{arg}{\text{max}}_{\rho \in T}(|T.\mathrm{CHILDREN}(\rho )\cap M|)$

13:  P←P∪{p}

14:  M←M∖T.CHILDREN(p) ◃ replace siblings with their parent

15:  **if**M=∅**then**

16:   SWAP(M,P) ◃ repeat on next level of taxonomic tree

17: **for**$t_{id}\in M\cup P$**do** ◃ output all assigned labels

18:  output(*r_id_*, *t_id_*)

In Algorithm 6, we follow a similar strategy for taxonomic label assignment as Centrifuge (Kim et al. 2016). Our algorithm takes an unordered sequence of taxonomic identifiers *L*, where each element in *L* is a triplet $\left(q_{id},t_{id},\ell \right)$ delivered by a call to label_assign in earlier stages. As before, *q_id_* is true if the match that triggered taxonomic assignment occurred with the forward version of the read, *t_id_* is the taxonomic identifier (label) of the reference string where the match occurred, and ℓ is the match’s length. Then, using information provided by the taxonomic tree T, we proceed as follows.

First, for the forward and reverse complement matches separately, we assign a score to each taxonomic label where a match occurred (lines 3-7). Here, following Centrifuge, we use $\ell_{\min}=15$. To aggregate the scores for each taxonomic label, we maintain two hash tables *H* and $\bar{H}$. We next identify the set *M* of all taxonomic labels that obtained the maximum score (line 9), which represents all labels that can potentially be assigned to read *r_id_*. To reduce |M| to within threshold *t_label_*, which denotes how many labels can be assigned to a single read (we use *t_label_* = 5 by default), we repeatedly replace the assigned labels that have the largest number of siblings in the taxonomic tree T with their parent in the tree (lines 11-16). Finally, the resulting labels are passed to output (lines 17-18).

## 5 Experimental results

To assess the performance of the CSBT and Coriolis, we performed a set of experiments using actual mobile platforms and MinION data. We ran all tests using the NVIDIA Jetson TX1 SCoC. This low-energy and small form-factor machine is equipped with a four-core ARMv8 processor and 4 GB of RAM, and is similar to the machine used in production by ONT in their MinIT and MinION Mk1C platforms. We stored all input data, including the reference databases, indexes, and input reads, on a Samsung 860 PRO 4 TB 2.5 Inch SATA III SSD attached to our machine via a SATA II bus. In this configuration, we measured the I/O bandwidth to be ∼250 MB/s. This is close to SATA II’s peak theoretical throughput of 300 MB/s. Our hardware setup ran Ubuntu 16.04.7 with the GNU/Linux 4.4.38-tegra kernel, and we compiled all evaluated software using g++ 9.4.0. In all reported experiments, we ensured that our hardware/software package used the performance governor to manage CPU cores (i.e. all cores operated at peak performance).

### 5.1 Test data

Our work on mobile DNA analytics is motivated by the need for *in situ* monitoring in remote locations of water pollution by algae. Therefore, in our tests we used a reference database of the relevant algae and bacterial genomes and performed MinION sequencing of algae mock communities. The resulting data, including the raw MinION reads, details of sequencing protocols, and identifiers of reference sequences, are available from SMARTEn (2023). A brief summary is provided below.

Our reference database consists of 1104 algae and bacterial genomes with 2 339 701 sequences spanning 12 255 Mbp, totaling to a size of ∼13 GB. To obtain input reads, we performed MinION sequencing of three mock communities. These communities consisted of five algal and two bacterial species (*Chlorella vulgaris*, *Scenedesmus obliquus*, *Chlamydomonas reinhardtii*, *Nannochloropsis oculata*, *Euglena gracilis*, *Aphanizomenon flosaque*, and *Synechocystis*) that frequently contaminate fresh water reservoirs and hence are of interest to environmental pollution monitoring. The resulting mock communities, which we will refer to as Even, HighC, and HighA, differed in their proportion of algal and bacterial species: Even contained an even concentration of DNA across all species while HighC and HighA contained a high concentration of bacterial and algae DNA, respectively. Here, a high concentration means that the resulting concentration of DNA was 100× higher than that of the other communities. We conducted the DNA sequencing following ONT’s recommended protocols for rapid sequencing using the R9.4 chemistry.

### 5.2 Reference database in external storage

The primary factor differentiating metagenomic classifiers is the type of text index used to manage the reference database. Hence, in the first set of experiments, we assessed how our CSBT compares to other popular text indexes when deployed in external storage on mobile setups. We focused primarily on the comparison between CSBTs and FM-index, since FM-index is considered the most efficient for carrying out exact queries. Additionally, we evaluated baseline SBT and suffix array implementations as additional reference points. Each index was constructed over our reference database. To query the indexes, we took a uniform random sample of 200 reads from the Even dataset, and then generated a set of queries following how Centrifuge and Coriolis implement match search. This resulted in 81 971 queries to each index.

For FM-index, we used the canonical implementation provided by the SDSL library (Gog et al. 2013), the state-of-the-art software for succinct data structures. SDSL is widely used in both industry and academia, comes packaged with most Linux distributions, and is a dependency of many other popular software tools, e.g. SeqAn3. Because SDSL does not support querying indexes from disk, we extended SDSL to memory map key building blocks, i.e. the succinct bit vectors that the actual indexes are comprised of. Additionally, we extended the main search algorithm for FM-index to search for the longest matching suffix instead of just exact matches. Our extensions are freely available and can be found at SMARTEn (2023).

For the suffix array, we used the construction algorithm provided by libdivsufsort (Mori 2022), which represents a suffix array as a plain array of 64-bit indexes complemented by the LCP array. We memory-mapped both arrays and implemented the classic binary search lookup algorithm.

For the CSBT, we used the implementation described in Section 4 with a node size of 48 KB. Finally, the SBT’s implementation was similar to that of the CSBT except it used the original representation and ignored duplicate suffixes. We found the SBT to be the most efficient in terms of the query throughput when using very large node sizes, so we used a node size of 512 KB. The sizes of the resulting indexes were 3 GB for FM-index, 207 GB for the suffix array, 361 GB for the SBT, and 169 GB for the CSBT.

**Figure 3.**
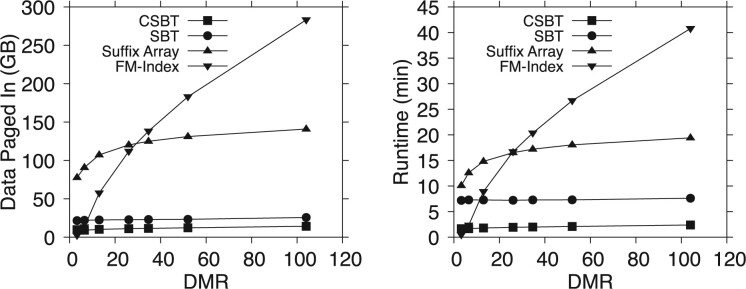
Query performance of each index for varying DMR.

For each index, we ran multiple tests varying the ratio between the reference data size and the main memory, which we refer to as the DMR. The DMR expresses how many times larger the reference data is than the available memory. To control the DMR we used Linux control groups (cgroups), a kernel-level mechanism for restricting the resources available to a process. We remounted the disk between each execution to ensure that all system-level caches were flushed. The results of the experiment are summarized in Table 1 and Fig. 3, where Table 1 shows the throughput of each index, and Fig. 3 the runtime of each index and the volume of data each index read from disk to run its queries.

**Table 1.** Throughput in queries/min for each index.

DMR	CSBT	SBT	Suffix Array	FM-Index
3.25	49 030.61	11 403.10	8125.46	173 606.07
6.5	49 274.25	11 259.72	6515.58	34 958.64
26	42 440.87	11 362.40	4964.46	4907.87
52	39 137.24	11 232.40	4542.90	3073.27
104	34 409.07	10 772.43	4223.27	2011.04

Our first observation is that while FM-index is the smallest and is efficient when it fits entirely into main memory (DMR 3.25), its performance drastically degrades as the DMR increases. Once as little as one third of the index must be accessed from external storage (DMR 6.5), we see that the CSBT outperforms FM-index, and the runtime of FM-index continues to grow rapidly. Even when as much as one third of FM-index still fits into main memory (DMR 13), the CSBT is already several times faster, and is over an order of magnitude faster once the reference data is 104× larger than the amount of available memory. Such a scenario is extremely plausible not only for mobile deployments but also for desktops and data centers, especially if we consider the entire NCBI database, which many applications desire to index.

The suffix array exhibits a similar trend, albeit less extreme, yet is still far slower than the CSBT while being larger than both the CSBT and FM-index, offering no practical advantage. In contrast, the SBT and CSBT maintain their performance as the DMR increases. Yet, the CSBT is several times faster than the SBT and has lower storage requirements.

From Fig. 3, we can see that the performance of each index directly correlates with its I/O intensity. Since CSBTs and SBTs are designed to minimize the number of I/O operations, they are resilient to increasing the DMR. On the other hand, FM-index, while very small, is extremely I/O intensive, primarily due to its inherent random memory accesses. This result is critical for mobile setups, as it implies that the CSBT is far more energy efficient than the other indexes. Unlike RAM memory, equipping a device with an idle SSD requires effectively no extra power. However, there is energy usage comparable to that of RAM drawn whenever a block of data is read from or stored on the disk. Hence minimizing I/O directly translates into lower power consumption.

The key takeaway from this experiment is that the CSBT exhibits stable and high performance irrespective of the amount of available memory, and outperforms the SBT in time and space. In fact, when the memory is significantly limited, the CSBT outperforms FM-index by over 16×. FM-index is the ideal solution when the entire index fits into main memory, but this is an infeasible assumption for realistic reference databases in mobile settings. The pace at which we can scale main memory is much slower than the exponential rate at which reference databases grow (Stephens et al. 2015). Furthermore, there are fundamental limits on the basis of cost and energy efficiency to how much main memory can be packed into a mobile device. On the other hand, even though the CSBT is much larger than FM-index (a factor of 56×), low-energy and high-capacity SSDs are inexpensive, readily available, and can be almost seamlessly coupled with the existing mobile setups.

### 5.3 Classification performance

In the second set of experiments, we compared Coriolis with the leading metagenomic classifiers when deployed on a portable SCoC. We focused on Kraken2 (Wood et al. 2019) and Centrifuge (Kim et al. 2016), which are used not only in metagenomic workflows but also in targeted nanopore sequencing (Payne et al. 2021). They are currently the only tools that can run in resource-constrained and ARM-based settings as they support memory-mapping of the reference databases. We also tested KrakenUnique (KrekenU) (Breitwieser et al. 2018), that although superseded by Kraken2, turns out to be worth considering as it differs from Kraken2 in how it stores the reference database. In the tests, we used KrakenU ver. 1.0.3, Kraken2 ver. 2.1.2, Centrifuge ver. 1.0.4, and our Coriolis implementation available at SMARTEn (2023).

We constructed each classifier’s database from the same dataset of our reference sequences. As in Section 5.2, we tested each classifier for varying DMR. This not only captures how the classifiers scale with the database size but also simulates how in real deployments the memory must be shared with other memory hungry tools, e.g. the basecaller, running on the device. We used the memory-mapping options of Centrifuge and the Kraken tools to enable out-of-core execution (otherwise neither tool would run). We executed KrakenU and Kraken2 with four threads and Centrifuge with two threads (since Centrifuge crashed when using any more). The number of threads used by Coriolis was adjusted dynamically by TBB, which usually assigns one thread per core. We tested each classifier with the first 30 minutes of sequencing data from each of the HighC, HighA, and Even datasets. We note that even with memory mapping enabled, Centrifuge was unable to run with DMR ≥ 6.5, and KrakenU was unable to run with a DMR of 52.

From Table 2, we can see that Coriolis significantly outperforms Centrifuge and is faster than both KrakenU and Kraken2 across all datasets and DMR factors. In fact, Coriolis is 10× faster than Centrifuge, while offering the same classification functionality. This happens because Centrifuge requires ∼20× more disk reads compared with Coriolis. KrakenU is the second best performing tool for lower DMR, but its performance degrades for higher DMR and is eventually incapable of running. To the contrary, Kraken2 can run with very high DMR, but this comes at significantly reduced performance. Kraken2 still experiences reduced throughput for higher DMR, and is much slower than KrakenU for small and mid-level DMR values. For both Kraken tools, the slowdown is caused by higher I/O, suggesting that, despite the use of well-optimized *k*-mer indexes, they cannot scale to much larger reference databases (i.e. a higher DMR).

**Table 2.** Classification throughput in kb/min for varying DMR.

	KrakenU			Kraken2			Centrifuge			Coriolis		
DMR	3.25	6.5	52	3.25	6.5	52	3.25	6.5	52	3.25	6.5	52
**HighC**	969.7	903.8	–	611.7	502.0	403.8	129.4	–	–	1323.8	1311.2	1251.2
**HighA**	1017.0	947.7	–	616.6	506.8	408.5	123.8	–	–	1273.1	1301.4	1213.0
**Even**	968.0	914.0	–	615.0	505.9	408.7	169.6	–	–	1287.7	1316.5	1243.7

On the other hand, the performance of Coriolis remains nearly identical across DMRs. This shows that Coriolis scales without relinquishing the advantages of string-based indexing. We also point out that while the databases of Kraken2 and Centrifuge are micro-optimized for storing NCBI genomic data, none of these optimizations has yet been applied to our CSBT implementation which powers Coriolis. Furthermore, in Section 6 we discuss several other optimizations that can improve Coriolis’s performance.

While the use of I/O optimal CSBTs is critical to maximize Coriolis’s efficiency, another key enabler is SMARTEn. By using SMARTEn and delegating all complex low-level details to its runtime, we rapidly prototyped Coriolis with just few lines of code. Moreover, SMARTEn’s automatic parallelism at runtime enables Coriolis to achieve far greater resource utilization than the other classifiers (we provide detailed insights in Supplement 1). Because SMARTEn separates I/O-bound tasks from compute-bound tasks and schedules each task individually, Coriolis makes progress even while tasks are waiting on I/O (i.e. match search) by switching execution to the compute-intensive tasks.

### 5.4 Classification effectiveness

In the last test, we evaluated effectiveness of each classifier using simulated reads generated with the NanoSim ver. 3.0.2 (Yang et al. 2017) from our three original datasets. NanoSim builds a statistical model of the sequencer directly from the actual reads, and the model accounts for the distribution of sequencing errors and reads length. The simulated reads come with the provenance data indicating from which genome and which position in the genome they have been derived. This information serves as the ground truth for classification. Moreover, the simulated reads exhibit the error rate and the length distribution akin to the original reads on which the NanoSim model has been trained. For each input dataset, we generated 100 000 reads such that the ratio of reads coming from the reference genomes reflected the species composition in the original samples (i.e. even number for Even, and 100× more algae reads than bacterial for HighA and vice versa for HighC). Next, we classified all the reads with each of the tested tools. Using the ground truth information attached to the reads, we assessed species-level sensitivity and species-level positive predictive value (PPV) as defined in Wood et al. (2019). Specifically, we define PPV as the ratio TP/(TP+FP), and sensitivity as TP/(TP+VP+FN+FP), where *TP* is the number of uniquely and correctly classified reads, *VP* is the number of vague reads (i.e. reads that have more than one label assigned, but one of them is correct), and *FP* and *FN* are, respectively, the number of incorrectly and unclassified reads.

From Table 3, we can see that Coriolis and Centrifuge are significantly outperforming KrakenU and Kraken2 in terms of PPV and accuracy. This confirms the earlier reports that classifiers based on exact pattern matching are better suited to MinION reads. The result is not entirely surprising considering that despite significant improvements, MinION reads still exhibit error rate at the level of ∼5%. The use of variable length exact matches by Coriolis and Centrifuge makes them more resilient to these errors. In our tests, the simulated reads had error rate of 5% to 17%, with median 13%. The higher than nominal error rate is something that one must expect during in the field deployments where sequencing may be affected by external conditions (e.g. temperature fluctuations). The lower effectiveness of all classifiers for HighC data is explained by the sample composition: the cyanobacteria in our tests turned out to be universally difficult to classify, and in HighC dataset the majority of reads come from these bacterial genomes.

**Table 3.** Classification effectiveness at species level.

		KrakenU	Kraken2	Centrifuge	Coriolis
**HighC**	Sensitivity	0.350	0.349	0.492	0.552
	PPV	0.366	0.365	0.557	0.581
**HighA**	Sensitivity	0.821	0.780	0.874	0.891
	PPV	0.891	0.789	0.984	0.985
**Even**	Sensitivity	0.456	0.551	0.575	0.661
	PPV	0.597	0.595	0.743	0.727

Overall, Centrifuge and Coriolis are comparable, with Coriolis offering a more balanced trade-off between sensitivity and precision. Although we would expect both classifiers to deliver the same result, they exhibit a slight variation due to minor differences in the label assignment algorithm.

## 6 Conclusions and future work

While our current work provides a fully functional prototype of a mobile metagenomic classifier that outperforms the state-of-the-art, there is still room for improvement. The limiting factor in this endeavor is waiting on I/O. To reduce the I/O bottleneck, we can apply system-level optimizations to CSBTs such as direct I/O, compression, and caching. Additionally, we could further extend SMARTEn to include abstractions for managing the reference database directly, enabling the SMARTEn runtime system to manage and optimize disk accesses in addition to computations. When a sufficiently stable network connection is available, the SMARTEn runtime can be easily extended to offload heavy computations (i.e. match search and match extend tasks) to remote edge servers with adequate resources for rapid processing. Such a runtime system could adaptively and transparently control the work distribution between the mobile device and the edge to optimize the utilization of both remote and local processing while protecting critical resources (e.g. energy) of a local device.

## Supplementary Material

btad243_Supplementary_Data

## Data Availability

All data are incorporated into the article and its online supplementary material and are available at http://score-group.org/?id=smarten.
