# Supplementary material for: Coriolis: enabling metagenomic classification on lightweight mobile devices
Source: Bioinformatics. 2023 Jun 30;39(Suppl 1):i66–75. doi: 10.1093/bioinformatics/btad243 (PMC10311295; doi:10.1093/bioinformatics/btad243)
Supplement: btad243_Supplementary_Data [file btad243_supplementary_data.pdf]

Coriolis: Enabling metagenomic classification on lightweight mobile devices (Supplement 1)

Andrew J. Mikalsen and Jaroslaw Zola

2023

1 Proofs

This section contains the proofs of the theorems from Section 3. For clarity, we restate Algorithms 1 and 2. First we will prove a useful property of the *lcp* function in Lemma 1.

Algorithm 1 Blind-Search(P)

$j \leftarrow 1$
$\ell \leftarrow 0$
for $i \leftarrow 2$ **up to** $|\mathcal{S}|$ **do**
    if $i - 1 = j$ **or** $LCP(i) \leq \ell$ **then**
        $\ell \leftarrow LCP(i)$
        if $\ell < |P|$ **and** $P(\ell + 1) = C_R(i)$ **then**
            $j \leftarrow i$
return j

Algorithm 2 Successor(P, ℓ)

$j \leftarrow$ Blind-Search(P)
$\ell' \leftarrow \ell + lcp\big(P[\ell+1..|P|], S_j[\ell+1..|S_j|]\big)$
if $|P| = \ell'$ **then**
    while $j > 1$ **and** $LCP(j) \geq \ell'$ **do**
        $j \leftarrow j - 1$
else
    $c \leftarrow P(\ell' + 1)$
    if $c < S_j(\ell' + 1)$ **then**
        while $j > 1$ **and** $LCP(j) \geq \ell'$
            and $\big(LCP(j) > \ell'$ **or** $c < C_L(j)\big)$ **do**
            $j \leftarrow j - 1$
    else
        $j \leftarrow j + 1$
        while $j \leq |\mathcal{S}|$ **and** $LCP(j) \geq \ell'$
            and $\big(LCP(j) > \ell'$ **or** $c > C_R(j)\big)$ **do**
            $j \leftarrow j + 1$
return (j, ℓ')

Lemma 1. *Given $A, B, C \in \Sigma^*$, if $lcp(A, B) < lcp(A, C)$, then $lcp(B, C) = lcp(A, B)$.*

Proof. We will show that by assuming $lcp(A, B) < lcp(A, C)$ we can derive $lcp(B, C) = lcp(A, B)$. Let $b = lcp(A, B)$ and $c = lcp(A, C)$. By the definition of *lcp*, we know that $A[1..b] = B[1..b]$ and $A[1..c] = C[1..c]$. Since $b < c$, we also have $A[1..b] = C[1..b]$. Thus, $B[1..b] = C[1..b]$, which implies that $lcp(B, C) \geq b$.

Next, we will show that $lcp(B,C) \leq b$. By definition, the longest common prefix between A and B is either (i) the shortest of the two strings or (ii) a proper prefix of both followed by a mismatch. In case (i) we assume $b = \min\{|A|,|B|\}$. Since $\min\{|A|,|B|\} = b < c \leq \min\{|A|,|C|\}$, we have $b = |B| < |A|$ and $|B| < |C|$. From the definition of lcp we have $lcp(B,C) \leq \min\{|B|,|C|\} = |B| = b$, so $lcp(B,C) \leq b$. In case (ii) we assume $b < \min\{|A|,|B|\}$ and $A(b+1) \neq B(b+1)$. Since $A[1..c] = C[1..c]$ and $0 \leq b < c$, we have $A(b+1) = C(b+1)$. By our assumption that $A(b+1) \neq B(b+1)$, we have $B(b+1) \neq C(b+1)$. Thus, by definition, $lcp(B,C) \leq b$. So in either case we have $lcp(B,C) \leq b$. Since $b \leq lcp(B,C) \leq b$, we have $lcp(B,C) = lcp(A,B)$. □

Theorem 1. BLIND-SEARCH(P) *returns* r *such that* $lcp(P,S_r) = \max\{lcp(P,S_k) \mid 1 \leq k \leq |\mathcal{S}|\}$.

Proof. The reader can verify that $LCP(i)$, $P(\ell+1)$, and $C_R(i)$ on lines 4, 5, and 6 are always defined and that the algorithm always terminates. We will proceed with a proof by contradiction. That is, we will assume $lcp(P,S_r) \neq \max\{lcp(P,S_k) \mid 1 \leq k \leq |\mathcal{S}|\}$ and derive false. Let $m = \max\{lcp(P,S_k) \mid 1 \leq k \leq |\mathcal{S}|\}$. Since $lcp(P,S_r) \neq m$, we must have $lcp(P,S_r) < m$. Because S is sorted lexicographically, there is some range $[b..e]$ with $1 \leq b \leq e \leq |\mathcal{S}|$ such that $k \in [b..e]$ if and only if $lcp(P,S_k) = m$. By our assumption, $r \notin [b..e]$, so we have the following two cases.

Case 1: $r < b$. Since $1 \leq r < b$, we have $b \geq 2$, implying that there's some iteration of the algorithm in which $i = b$. Consider the beginning of this iteration. We will first show that the if statement on line 4 holds. This is clearly the case when $b-1 = j$, so consider instead the case when $b-1 \neq j$. We must have $b > 2$, since otherwise we'd have $b = 2$ and $j = 1$ contrary to our assumption. Because $b > 2$, there must be some most recent prior iteration where $i = i'$ for some $2 \leq i' < b$ in which ℓ was assigned its current value $LCP(i')$ on line 5. Observe that we must have $LCP(i') \geq |P|$ or $C_R(i') \neq P(\ell+1)$. To demonstrate this, assume for the purpose of contradiction that $LCP(i') < |P|$ and $C_R(i') = P(\ell+1)$. Then we'd assign i' to j on line 7. Since by the definition of i' line 7 isn't executed again until at least iteration $i = b$, we have $j = i'$ which implies $i' < b-1$ by our assumption that $j < b-1$. This means there is some iteration after i' but before b. But then during the iteration after i', the if condition on line 4 would hold and we'd assign $LCP(i'+1)$ to ℓ, contradicting the definition of i'. Thus, we have $LCP(i') \geq |P|$ or $C_R(i') \neq P(\ell+1)$. In the first case where $LCP(i') \geq |P|$, we know from the definition of b that $lcp(P,S_{b-1}) < lcp(P,S_b)$. By Lemma 1 we have $LCP(b) = lcp(P,S_{b-1})$. And since $lcp(P,S_b) \leq |P|$, we can conclude that $LCP(b) \leq |P|$, and thus $LCP(b) \leq \ell$. In the second case where $C_R(i') \neq P(\ell+1)$, observe that by the definition of i', we must have the if condition on line 4 be false for every iteration after i' and before b. Thus, for all $i' < k < b$, $LCP(k) > LCP(i')$. This implies that $S_{i'}[1..LCP(i')+1] = S_k[1..LCP(i')+1]$ for all $i' < k < b$. That is, $S_{i'}, S_{i'+1}, \ldots, S_{b-1}$ all share the same prefix of length $LCP(i')+1$. Again by Lemma 1, $lcp(S_{i'},S_b) = lcp(P,S_{i'}) < lcp(P,S_b)$, which implies $S_b(\ell+1) = P(\ell+1)$. Thus, we have $S_{b-1}(\ell+1) = S_{i'}(\ell+1) = C_R(i') \neq P(\ell+1) = S_b(\ell+1)$, meaning that $LCP(b) \leq \ell$ by the definition of lcp. This shows that the if statement on line 4 holds, so we execute lines 5 and 6. Now we have $\ell = LCP(i)$. Since $lcp(P,S_{b-1}) < lcp(P,S_b) \leq |P|$, we have $\ell = lcp(P,S_{b-1})$ by Lemma 1, so $\ell < |P|$. Also, by $\ell < lcp(P,S_b)$, we have $P(\ell+1) = S_b(\ell+1) = C_R(b)$. Thus, the if statement on line 6 holds and we assign b to j. But since j is monotonically increasing and $r < b$, this means we don't return r, contradicting our assumption that r is returned.

Case 2: $e < r$. Since S is lexicographically ordered, each of $S_e, S_{e+1}, \ldots S_r$ share the same prefix of length $lcp(S_e,S_r)$. By definition, we have $lcp(P,r) < lcp(P,e)$, which by Lemma 1 implies that $lcp(e,r) = lcp(P,r) < lcp(P,e)$. Thus we have $S_e(lcp(e,r)+1) = P(lcp(e,r)+1) \neq S_r(lcp(e,r)+1)$ since S is prefix free. Let k be the first index in $[e+1..r]$ such that $S_e(lcp(S_e,S_r)+1) \neq S_k(lcp(S_e,S_r)+1)$. We will show inductively that at the end of every iteration of the for loop where $i \in [k..r]$ we maintain that $j < k$ and that $\ell = lcp(e,r)$. When $i = k$, we have $j < k$ at the start of the iteration. By definition $e < k \leq r$ and thus we have $LCP(k) = lcp(S_e,S_r)$, meaning the if condition on line 4 is satisfied and we assign $lcp(S_e,S_r)$ to ℓ, maintaining the invariant on ℓ. By definition, we have $S_k(LCP(k)) \neq S_e(LCP(k)) = P(LCP(k))$, so $C_R(k) \neq P(LCP(k))$ and we don't execute line 7, maintaining that $j < k$. Now assume the invariant held for $i-1 \in [k..r]$, and we are now at the beginning of the next iteration where $i \in [k..r]$. If $LCP(i) > \ell$ then we do nothing, and the invariant is trivially maintained. Since all suffixes in the range share a prefix of length ℓ, the only other possibility is $LCP(i) = \ell$. Thus, assigning $LCP(i)$ to ℓ still maintains the invariant. By lexicographic order, we must have $S_i(\ell+1) > S_e(\ell+1)$, which implies $S_i(\ell+1) \neq P(\ell+1)$. As a result,

we again do not execute line 7, and maintain that $j < k$. Now that we've shown the invariant is maintained, consider the iteration when $i = r$. Since by our invariant we have $j < k$ at the end of the iteration, and since j is only ever assigned the current value of i, which is strictly increasing, this means that we don't return r, contradicting our assumption.

Since we arrive at a contradiction in either case, we must have $lcp(P, S_r) = \max\{lcp(P, S_k) \mid 1 \leq k \leq |\mathcal{S}|\}$. □

Theorem 2. *For alphabet Σ, given $P \in \Sigma^*$ and $\ell \in \mathbb{N} \cup \{0\}$ such that no $S_i \in S$ is a prefix of P and $\ell \leq \max\{lcp(P, S_k) \mid 1 \leq k \leq |\mathcal{S}|\}$, SUCCESSOR$(P, \ell)$ returns (j, ℓ') such that $j = \min\{1 \leq k \leq |\mathcal{S}| + 1 \mid k > |\mathcal{S}| \vee P \leq S_k\}$ and $\ell' = \max\{lcp(P, S_k) \mid 1 \leq k \leq |\mathcal{S}|\}$.*

Proof. By Theorem 1, we have $lcp(P, S_j) = \max\{lcp(P, S_k) \mid 1 \leq k \leq |\mathcal{S}|\}$. Observe that by our assumptions we have $\ell' = lcp(P, S_j) = \max\{lcp(P, S_k) \mid 1 \leq k \leq |\mathcal{S}|\}$ after assigning ℓ' on line 2. By the definition of lcp and since S_j isn't a prefix of P, we have the following three cases.

Case 1: $|P| = \ell'$. In this case we execute the while loop on line 4. Since the algorithm decrements j every iteration and terminates when $j < 1$, the loop always terminates. We will show that before every iteration of the loop we maintain that $lcp(P, S_j) = \ell'$. By the definition of ℓ' this is clearly true before the first iteration. Now assume the invariant holds before the start of an iteration. If the loop doesn't terminate, then we have $LCP(j) \geq \ell'$, which implies that $lcp(P, S_{j-1}) \geq \ell'$ by our assumption. And by the definition of ℓ', this means $lcp(P, S_{j-1}) = \ell'$. Since we then decrement j, we then have $lcp(P, S_j) = \ell'$ before the next iteration. Thus, when the loop terminates, we have $lcp(P, S_j) = \ell'$. Since $lcp(P, S_j) = \ell' = |P|$, we have that P is a prefix of S_j, thus $P \leq S_j$. By the termination condition, we have either $j = 1$ or $LCP(j) < \ell'$. If $j = 1$, since $P \leq S_j$, clearly we have $j = \min\{1 \leq k \leq |\mathcal{S}| + 1 \mid k > |\mathcal{S}| \vee P \leq S_k\}$. If $LCP(j) < \ell'$, then by the fact that S is lexicographically ordered, we have that $S_{j-1} < P$, thus again $j = \min\{1 \leq k \leq |\mathcal{S}| + 1 \mid k > |\mathcal{S}| \vee P \leq S_k\}$.

Case 2: $|P| > \ell' \wedge P(\ell' + 1) < S_j(\ell' + 1)$. In this case we execute the while loop on line 9. Again, it's easy to see that the while loop always terminates. We will show that before every iteration of the loop we maintain that $lcp(P, S_j) = \ell'$ and $c < S_j(\ell' + 1)$. By the definitions of ℓ' and c along with our assumption, this is clearly true before the start of the first iteration. Now assume the invariant holds before the start of an iteration. If the loop doesn't terminate, then we have $LCP(j) \geq \ell'$. By our assumption, this implies that $lcp(P, S_{j-1}) \geq \ell'$, which by the definition of ℓ' means that $lcp(P, Sj - 1) = \ell'$. We also have that $LCP(j) > \ell'$ or $c < C_L(j)$. Consider the cases (i) $LCP(j) > \ell'$ and (ii) $LCP(j) = \ell'$. In case (i), we have that $S_{j-1}(\ell' + 1) = S_j(\ell' + 1)$, and since by our assumption $c < S_j(\ell' + 1)$, we have that $c < S_{j-1}(\ell'+1)$. In case (ii), we must have that $c < C_L(j)$. Since $LCP(j) = \ell'$, we have $C_L(j) = S_{j-1}(\ell'+1)$, and thus $c < S_{j-1}(\ell' + 1)$. So in either case $c < S_{j-1}(\ell' + 1)$. Since we then decrement j, we then have $lcp(P, S_j) = \ell'$ and $c < S_j(\ell' + 1)$ before the next iteration. Because $lcp(S_j) = \ell'$ and $c < S_j(\ell' + 1)$, $P < S_j$. By the termination condition, we have either $j = 1$, $LCP(j) < \ell'$, or $LCP(j) = \ell' \wedge c > C_L(j)$. If $j = 1$, since $P \leq S_j = S_1$, clearly we have $j = \min\{1 \leq k \leq |\mathcal{S}| + 1 \mid k > |\mathcal{S}| \vee P \leq S_k\}$. If $LCP(j) < \ell'$, then by the fact that S is lexicographically ordered, we have that $S_{j-1} < P$, thus again $j = \min\{1 \leq k \leq |\mathcal{S}| + 1 \mid k > |\mathcal{S}| \vee P \leq S_k\}$. Finally, if $LCP(j) = \ell'$ and $c > C_L(j)$, we must still have $S_{j-1} < P$, implying $j = \min\{1 \leq k \leq |\mathcal{S}| + 1 \mid k > |\mathcal{S}| \vee P \leq S_k\}$.

Case 3: $|P| > \ell' \wedge P(\ell' + 1) > S_j(\ell' + 1)$. In this case, we execute the while loop on line 13. Since the algorithm increments j every iteration and terminates when $j = |\mathcal{S}| + 1$, the loop always terminates. We will show that before every iteration of the loop we maintain that $lcp(P, S_{j-1}) = \ell'$ and $S_{j-1}(\ell' + 1) < c$. By the definitions of ℓ' and c along with our assumption, this is clearly true before the start of the first iteration. Now assume the invariant holds before the start of an iteration. If the loop doesn't terminate, then we have $LCP(j) \geq \ell'$. By our assumption, this implies that $lcp(P, S_j) \geq \ell'$, which by the definition of ℓ' means that $lcp(P, S_j) = \ell'$. We also have that $LCP(j) > \ell'$ or $c > C_R(j)$. Consider the cases (i) $LCP(j) > \ell'$ and (ii) $LCP(j) = \ell'$. In case (i), we have that $S_{j-1}(\ell' + 1) = S_j(\ell' + 1)$, and since by our assumption $S_{j-1}(\ell' + 1) < c$, we have that $S_j(\ell' + 1) < c$. In case (ii), we must have that $c > C_R(j)$. Since $LCP(j) = \ell'$, we have $C_R(j) = S_j(\ell' + 1)$, and thus $S_j(\ell' + 1) < c$. So in either case $S_j(\ell' + 1) < c$. Since we increment j, we then have $lcp(P, S_{j-1}) = \ell'$ and $S_{j-1}(\ell' + 1) < c$ before the next iteration. Because $lcp(P, S_{j-1})$ and $S_{j-1}(\ell' + 1) < c$, $P < S_j$. By the termination condition, we have either $j = |\mathcal{S}| + 1$, $LCP(j) < \ell'$, or

$LCP(j) = \ell' \wedge c < C_R(j)$. If $j = |\mathcal{S}| + 1$, since S is sorted lexicographically and $S_{j-1} = S_{|\mathcal{S}|} < P$, there is no S_k for which $P \leq S_k$. Thus, we have $j = |\mathcal{S}| + 1 = \min\{1 \leq k \leq |\mathcal{S}| + 1 \mid k > |\mathcal{S}| \vee P \leq S_k\}$. If $LCP(j) < \ell'$, then because S is lexicographically ordered we have $S_{j-1} < P \leq S_j$, which implies that $j = \min\{1 \leq k \leq |\mathcal{S}| + 1 \mid k > |\mathcal{S}| \vee P \leq S_k\}$. Finally, if both $LCP(j) = \ell'$ and $c < C_R(j)$, we must still have $S_{j-1} < P \leq S_j$, implying $j = \min\{1 \leq k \leq |\mathcal{S}| + 1 \mid k > |\mathcal{S}| \vee P \leq S_k\}$.

Therefore, in every case, we return (j, ℓ') where $j = \min\{1 \leq k \leq |S| + 1 \mid k > |S| \vee P \leq S_k\}$ and $\ell' = \max\{lcp(P, S_k) \mid 1 \leq k \leq |S|\}$, which completes the proof. □

Theorem 3. *Assume that $\mathcal{S}$, LCP, C_L, and C_R reside in internal memory and that each $S_i \in \mathcal{S}$ is represented as a pointer to some string stored in external memory. Given $P \in \Sigma^*$ such that no $S_i \in S$ is a prefix of P and $\ell \leq \max\{lcp(P, S_k) \mid 1 \leq k \leq |S|\}$, $\textsc{Successor}(P, \ell)$ takes $O\left(\frac{|P|-\ell}{B}\right)$ I/Os.*

Proof. The only operation that performs any I/Os is computing ℓ' on line 2 by loading characters of $S_j\left[\ell + 1..|S_j|\right]$ and $P\left[\ell + 1..|P|\right]$. This computation loads at most $2 \cdot \left|P\left[\ell + 1..|P|\right]\right|$ characters (for both P and S_j), and since $\left|P\left[\ell + 1..|P|\right]\right| = |P| - \ell - 1$, the I/O complexity is $O\left(\frac{|P|-\ell}{B}\right)$. □

2 Coriolis Runtime Characteristics

Here we report additional measurements taken while comparing the different classifiers. Table S1 compares each of Kraken2, Centrifuge, and Coriolis while varying the number of available cores. We note that Centrifuge was unable to run with more that two threads. Table S2 provides statistics reported by `sar` during the experiments from Section 5.3.

In Table S1, we observe that the speedup for Kraken2 and Centrifuge is negligible, while Coriolis achieves reasonable speedup for two cores, which begins to drop off once reaching four cores. Even with a single core, Coriolis is almost at the speed of Kraken2. Once using two cores, Coriolis is nearly twice as fast as Kraken2 and over four times faster than Centrifuge, and still achieves non-negligible speedup when increasing to four cores. It is worth reiterating that our implementation of Coriolis makes no effort to parallelize the classifier. Instead, simply by expressing Coriolis in our programming model, the SMARTEn runtime automatically parallelizes Coriolis' execution. With this in mind, the scaling of Coriolis is quite impressive, and demonstrates the power of the SMARTEn programming model.

In Table S2, we see the impact of I/O efficiency and CPU utilization on the speed of classification. Our first observation is that for each classifier, the user CPU time (that is, the total execution time spent utilizing CPU in the user-space) remains effectively constant across different DMRs. The small variations present (in the order of seconds) can be easily attributed to noise in the system once considering that the tools ran for multiple hours. The system CPU time (that is, the total execution time spent by the kernel using the CPU) similarly remains relatively fixed. We can see that the system CPU time dominates the user CPU time. This is because the I/O intensity is quite significant, with each classifier needing to load terabytes of data over the course of execution. Because each of these classifiers use memory mapping, the high I/O intensity puts pressure on the kernel to manage the memory-mapped pages. We see that the total amount of data read from disk by Centrifuge is significantly larger than Kraken2 and Coriolis, explaining why Centrifuge is substantially slower. We see that increasing the DMR has a far greater impact on I/O intensity for Kraken2 than it does for Coriolis, explaining why Coriolis is faster than Kraken2 for a high DMR.

Table S1: Runtime, speedup, and average CPU utilization for each classifier.

	Number of Threads	**1**	**2**	**4**
**Kraken2**	Runtime (sec)	154	151	150
	Speedup	1.00	1.02	1.03
	Idle CPU (%)	73.93	73.27	73.07
**Centrifuge**	Runtime (sec)	482	435	–
	Speedup	1.00	1.11	–
	Idle CPU (%)	73.49	70.27	–
**Coriolis**	Runtime (sec)	181	107	96
	Speedup	1.00	1.69	1.89
	Idle CPU (%)	74.08	70.06	59.89

Table S2: Runtime and I/O characteristics of each classifier.

		**HighCyano**		**HighAlgae**		**AllEven**	
DMR		3.5×	6.5×	3.5×	6.5×	3.5×	6.5×
Kraken2	Data Paged In (GB)	2,335.56	2,859.40	5,183.47	6,348.88	2,370.94	2,900.61
	System CPU Time (min)	13.08	14.03	28.99	30.98	13.28	14.09
	User CPU Time (min)	0.27	0.39	0.64	0.66	0.28	0.32
Centrifuge	Data Paged In (GB)	9,492.17	–	22,560.99	–	7,323.17	–
	System CPU Time (min)	55.83	–	130.81	–	44.45	–
	User CPU Time (min)	1.86	–	3.36	–	1.28	–
Coriolis	Data Paged In (GB)	2,568.00	2,622.06	5,821.20	5,966.64	2,542.72	2,603.94
	System CPU Time (min)	15.50	13.77	35.77	31.73	15.41	13.82
	User CPU Time (min)	11.32	11.32	26.73	26.60	11.73	11.75
